# Features of lexical complexity: insights from L1 and L2 speakers

**DOI:** 10.3389/frai.2023.1236963

**Published:** 2023-11-30

**Authors:** Kai North, Marcos Zampieri

**Affiliations:** School of Computing, George Mason University, Fairfax, VA, United States

**Keywords:** readability, text simplification, language acquisition, educational technology, complex word identification (CWI)

## Abstract

We discover sizable differences between the lexical complexity assignments of first language (L1) and second language (L2) English speakers. The complexity assignments of 940 shared tokens without context were extracted and compared from three lexical complexity prediction (LCP) datasets: the CompLex dataset, the Word Complexity Lexicon, and the CERF-J wordlist. It was found that word frequency, length, syllable count, familiarity, and prevalence as well as a number of derivations had a greater effect on perceived lexical complexity for L2 English speakers than they did for L1 English speakers. We explain these findings in connection to several theories from applied linguistics and then use these findings to inform a binary classifier that is trained to distinguish between spelling errors made by L1 and L2 English speakers. Our results indicate that several of our findings are generalizable. Differences in perceived lexical complexity are shown to be useful in the automatic identification of problematic words for these differing target populations. This gives support to the development of personalized lexical complexity prediction and text simplification systems.

## 1 Introduction

A growing body of research has focused on the detection of complex words for automatic text simplification (TS) (Paetzold and Specia, 2016; Yimam et al., 2018; Shardlow et al., 2021a). Complex words within TS frameworks are those words that are difficult to recognize, understand, or articulate and can significantly reduce reading comprehension (Kyle et al., 2018; Shardlow et al., 2021a). Perceived lexical complexity is therefore the level of difficulty associated with any given word form by a particular individual or group.

First language (L1) English speakers find certain words to be more or less complex than second language (L2) English speakers. This may be due to differing degrees of familiarity (Shardlow et al., 2021b), L1 influence on L2 production (Lee and Yeung, 2018b; Maddela and Xu, 2018), greater cognitive load in L2 processing (McDonald, 2006; Hopp, 2014), differences between L1 and L2 lexical and syntactic encoding and activation (Clahsen and Felser, 2006a,b, 2018), and various other phenomena (Crossley and McNamara, 2009).

With distance learning becoming ever more popular (Morris et al., 2020), research has been focusing on identifying barriers and improving approaches to online education (McCarthy et al., 2022). This includes an increase in the demand for AI/NLP technologies such as TS that can be utilized within computer-assisted language learning (CALL) applications (Tseng and Yeh, 2019; Rets and Rogaten, 2020). However, little research has been conducted on the similarities or differences between L1 and L2 English speakers' perception of lexical complexity in the field of automatic lexical complexity prediction. Demographic differences between annotators hinder the automatic detection of complex words, and in turn reduce the performance of generalized TS technologies (Zeng et al., 2005; Lee and Yeung, 2018b; Maddela and Xu, 2018). As far as the authors are aware, this correlation has not been fully explored within complexity prediction literature, especially in consideration with theoretical explanations from applied linguistics (Zeng et al., 2005; Tack et al., 2016; Lee and Yeung, 2018b; Shardlow et al., 2021a,b; Tack, 2021).

To aid complexity prediction research along with its downstream TS and CALL applications, this study asks the following research questions:

- **RQ 1:** Are there sizable differences between L1 and L2 English speakers perception of lexical complexity reflected in the annotation of existing complexity prediction corpora?
- **RQ 2:** If these differences exist, are they represented by the features commonly used in systems developed for complexity prediction?

Section 2 introduces the reader to complexity prediction research. Section 2.2 details several datasets used for complexity prediction. Section 2.3 provides features related to lexical complexity. Section 4 draws several conclusions regarding the strength of the correlations between these features and the complexity assignments of L1 and L2 English speakers. Section 5 gives several brief possible explanations taken from applied linguistics. Section 6 lastly shows the application of our findings for automatically classifying spelling errors made by L1 and L2 English speakers. This was done to test the validity of our findings and to provide a potential use case within TS and CALL technologies.

## 2 A survey of existing datasets and features

### 2.1 Complexity prediction research

Automatic complexity prediction is primarily split into (1) complex word identification, and (2) lexical complexity prediction. Complex word identification (CWI) entails the development of binary classifiers that can automatically distinguish between complex and non-complex words. They achieve this by assigning target words with binary complexity values of either 0 (non-complex) or 1 (complex) (Table 1) (Paetzold and Specia, 2016; Yimam et al., 2018). Lexical complexity prediction (LCP) is essentially a regression based task. It relies on multi-labeled data to model lexical complexity on a continuum. This continuum has varying thresholds, which may range from very easy (0), easy (0.25), neutral (0.5), difficult (0.75), to very difficult (1). These thresholds are used to label a target word with a continuous complexity value between 0 and 1 (Table 1). LCP, therefore, provides more fine-grained complexity values than in comparison to the binary annotated data provided by CWI, as it is able to recognize those words with a neutral level of complexity (Shardlow et al., 2020, 2021a). This study has subsequently focused on LCP, hence continuous complexity assignments made by L1 and L2 English speakers.

**Table 1:** Example of a sentence annotated with both binary and continuous complexity values from CWI and LCP systems, respectively, taken from the CompLex dataset (Shardlow et al., 2020).

**Extract:**	**Folly**	**is**	**set**	**in**	**great dignity**
**Binary Complexity:**	1	is	0	in	0
**Continuous Complexity:**	0.57	is	0.18	in	0.15

1 is complex and 0 is non-complex. Target words are in bold.

Researchers interested in both CWI and LCP have brought into question the generalizability of automated lexical complexity assignments. They argue that prior CWI and LCP systems are unable to account for “variations in vocabulary knowledge among their users” (Lee and Yeung, 2018b). In other words, they are unable to account for differing perceptions of lexical complexity. Studies, such as Zeng et al. (2005), Tack et al. (2016), Lee and Yeung (2018b), and Tack (2021), introduced personalized CWI to account for such variation among differing target populations. Personalized CWI caters for the individual by taking into consideration their specific demographic and by relying on features that correlate with that demographic's assignment of lexical complexity.

Traditionally, research has indicated statistical and psycholinguistic features as being reliable indicators of a word's complexity (Shardlow et al., 2021b), such as word frequency, length, familiarity and concreteness. Nevertheless, since lexical complexity assignments differ from one demographic to the next, the question remains whether these features are truly universal in their ability to predict lexical complexity across multiple target populations. These features include word frequency, word length, syllable count, familiarity, prevalence, and concreteness (Paetzold and Specia, 2016; Monteiro et al., 2023). Thus, to answer research questions 1 and 2, numerous LCP datasets have been selected to represent the lexical complexity assignments of both L1 and L2 English speakers (Section 2.2). The above features were then applied to these datasets to uncover whether sizable differences exist between each set of annotators' complexity assignments, as well as these features ability to predict lexical complexity for each target demographic (Section 4).

### 2.2 Existing datasets

Shared-tasks have increased the popularity of complexity prediction research (Paetzold and Specia, 2016; Yimam et al., 2018; Shardlow et al., 2021a). This has resulted in several datasets that can be used for complexity prediction. These datasets have been labeled by annotators from differing backgrounds. Some datasets were created by annotators made up of purely L1 English speakers or annotators from a specific country, such as China (Lee and Yeung, 2018a; Yeung and Lee, 2018), Japan (Nishihara and Kajiwara, 2020), or Sweden (Smolenska, 2018). Other datasets contained a mixture of L1 and L2 English speakers from a variety of international backgrounds (Yimam et al., 2018). However, few of these datasets consist of multi-labeled continuous data used to train state-of-the-art LCP systems. In fact, only several datasets exist that contain English words annotated using a likert-scale and labeled with continuous complexity values. Examples include the CompLex dataset (Shardlow et al., 2020), the Word Complexity Lexicon (Maddela and Xu, 2018), and the CERF-J project's word list (Tono, 2017). These datasets have been grouped in accordance to their annotators and have been described throughout the following sections.

#### 2.2.1 Dataset with L1 English speaking annotators

The CompLex dataset (Shardlow et al., 2020) is the most recent dataset that has been used to develop LCP systems (Shardlow et al., 2020, 2021a). Over 1500 L1 English speaking annotators were responsible for labeling continuous complexity values to a range of extracts taken from the Bible (Christodouloupoulos and Steedman, 2015), biomedical articles (Koehn, 2005) and Europarl (Bada et al., 2012). These annotators were crowd-sourced from “the UK, USA, and Australia” (Shardlow et al., 2020). Having been sourced from English-speaking countries, it is likely that these annotators were predominately L1 English speakers. The CompLex dataset is also split into two sub-datasets. The first contains 9,000 instances of single words. The second houses 1,800 instances of multi-word expressions (MWEs). Both sub-datasets were created using a 5-point likert scale and therefore provide continuous complexity values ranging between 0 (very easy) and 1 (very difficult). Assigned complexity values were averaged. The returned averaged values were then used as the corresponding target words', or MWEs', overall level of complexity.

#### 2.2.2 Datasets with L2 English speaking annotators

The Word Complexity Lexicon (WCL) (Maddela and Xu, 2018) consists of “15,000 English words with word complexity values assessed by human annotators” (Maddela and Xu, 2018). These annotators were 11 non-native yet fluent L2 English speakers from varying international backgrounds. These annotators also had varying first languages. The WCL contained the most frequent 15,000 words provided by the Google 1T Ngram Corpus (Brants and Franz, 2006). Its complexity values were continuous and were gained through the use of a 6-point likert scale. Each annotator was asked whether they believed the target word was either very simple, moderately simple, simple, complex, moderately complex, or very complex.

The Common European Reference Framework for Languages (CERF) is a recognized criteria for assessing language ability. It contains multiple levels. These levels range from: “A1 (elementary), A2, B1, B2, C1, to C2 (advanced)” (Uchida et al., 2018). A1 is used to refer to elementary proficiency, having the ability to “recognise familiar L2 words and very basic phrases” (Council of Europe, 2020). C2 denotes advanced proficiency, “having no difficulty in understanding any kind of L2 [spoken or written] language” (Council of Europe, 2020). The CERF-J project is the utilization of the CERF for English foreign-language teaching in Japan. The project contains a CERF-J wordlist with 7800 English words, with each word having been assigned a CERF level marking their complexity. These assigned CERF levels were calculated in accordance to a word's frequency within CERF rated foreign-language English textbooks. These textbooks were taken from Chinese, Taiwanese, and Korean schools (Markel, 2018; Tono, 2017). As such, the complexity assignments contained within the CERF-J wordlist may reflect those made by Chinese, Taiwanese, or Korean L2 English speaking annotators.

#### 2.2.3 Datasets with L1 and L2 English speaking annotators

The Personalized LS Dataset was created by Lee and Yeung (2018b) to reflect the individual complexity assignments of 15 Japanese learners of English. These L2 English speakers were tasked with rating the complexity of 12,000 English words. To do so, they used a 5-point likert scale that depicted how well they knew the word. They chose from 5 labels that ranged from (1) “never seen the word before”, to (5) “absolutely know the word's meaning” (Lee and Yeung, 2018b). The dataset classified those words labeled between 1 and 4 as being complex, whereas those labeled 5 were believed to be non-complex. Regardless of this binary classification, the use of a 5-point likert scale means that such data can easily be adapted for continuous LCP rather than for binary CWI. The annotators of the Personalized LS Dataset were also sub-divided in regards to their English proficiency (Lee and Yeung, 2018b). The first sub-group contained the four least proficient annotators whom knew less than 41% of the 12,000 English words. The second sub-group consisted of the four most proficient annotators whom knew more than 75% of the 12,000 English words. Unfortunately, however, the Personalized LS Dataset is not publicly available and was therefore not used within this study.

The CWI–2018 shared-task (Yimam et al., 2018), introduced several participating teams to a set of CWI datasets annotated by a variety of L1 and L2 English speaking annotators. These datasets were of differing genres containing extracts taken from news articles, Wikinews and Wikipedia. These datasets were also of differing languages, being English, German, Spanish and French. Its annotators were collected using the Amazon Mechanical Turk (MTurk) and were tasked with identifying complex words from a given number of extracts (Yimam et al., 2018). In total, 134 L1 and 49 L2 English speakers labeled the English datasets with 34,789 binary complexity values. Due to CWI–2018's use of annotators from a variety of backgrounds, as well as its datasets being constructed from multiple sources, the CWI–2018 datasets acted as a good control during our initial analysis by indicating which of our selected features (Section 2.3) were salient across both sets of annotators. However, due to the CWI–2018 datasets containing binary instead of continuous complexity assignments, these datasets were later dropped. This is since a direct comparison between binary complexity values and continuous complexity values is less informative and is subsequently less helpful in the development of state-of-the-art LCP systems that rely on continuous data.

### 2.3 Features

Many CWI and LCP classifiers use statistical, phonological, morphological, and psycholinguistic features to predict lexical complexity (Shardlow et al., 2021b). Among these features, word frequency, word length, syllable count, and familiarity are the most common (Paetzold and Specia, 2016; Yimam et al., 2018; Shardlow et al., 2021b). Despite current state-of-the-art LCP systems preferring the adoption of unsupervised deep learning transformer-based models (Pan et al., 2021; Rao et al., 2021; Yaseen et al., 2021), those LCP systems that rely on feature engineering still perform well. During the LCP–2021 shared-task (Shardlow et al., 2021a), the third best performing system adopted a feature engineering approach (Mosquera, 2021). Among Mosquera (2021)'s extensive set of features, word frequency, length, syllable count, and familiarity were found to be among the best features in predicting lexical complexity. This is further supported by the findings of Desai et al. (2021) and Shardlow et al. (2021b). As such, this study has used these features as a means of analyzing the differences in complexity assignment between L1 and L2 English speakers.

#### 2.3.1 Statistical features

Zipf's Law states that few words are rare, few words are very frequent, and the rest are more or less evenly distributed. Those words which are rare and that appear less frequently within a text are likely to be longer than compared to those words that are more common (Quijada and Medero, 2016; Zampieri et al., 2016). Therefore, it is often believed that since infrequent words are longer, they are less likely to be familiar and as a consequence, are more complex than compared to more frequent words that are shorter (Zampieri et al., 2016).

Zipfian frequency is used to predict the frequency of a target word within a natural language, such as English, given a provided dataset. It is calculated per the following equation:


(1) $$\begin{array}{l} ZipfFreq\left(word\right)=\frac{1}{k^{s}H_{n,s}}=\frac{1}{k_{word}} \end{array}$$

where *k* is the frequency rank of the target word ordered from the most to least frequent, *s* is the exponent that defines the distribution, *n* is the vocabulary, and size *H*_*n, s*_ is the generalized harmonic number; “being the sum of the reciprocals of the size of the vocabulary” (Zampieri et al., 2016).

True frequency represents the frequency of a target word within a given dataset rather than its predicted frequency within its respective language. True frequency is generated through the following equation:


(2) $$\begin{array}{l} TrueFreq\left(word\right)=\frac{count\left(word\right)}{N} \end{array}$$

with the numerator being the number of times the target word appeared in a dataset, and where N is the number of tokens within that dataset. We calculated frequency using the Brown Corpus (Francis and Kucera, 1979) and the British National Corpus (BNC) (BNC Consortium, 2015).

A percentage of the BNC was used to generate document frequencies, being how many documents the target word was found in. The BNC consists of 4049 texts, including both written and spoken texts (BNC Consortium, 2015). We selected a percentage of written texts, with an average of 10% of our selected texts coming from each text genre, spanning literary works to news and scientific articles. We believed document frequency would help verify or disprove any potential correlation drawn between lexical complexity and word frequency.

Word length is associated with lexical complexity (Paetzold and Specia, 2016; Yimam et al., 2018; Desai et al., 2021; Shardlow et al., 2021a,b). It is calculated by simply counting the number of characters that form a target word. Zampieri et al. (2016) along with others (Paetzold and Specia, 2016; Yimam et al., 2018; Desai et al., 2021; Shardlow et al., 2021a,b), have discovered that statistical features, such as word frequency, be it either Zipfian frequency or True frequency, along with word length, are good baseline indicators of lexical complexity. A strong negative correlation should be seen between word frequency, word length and complexity, regardless of whether the annotator is a L1 or L2 speaker.

#### 2.3.2 Phonological features

Syllable count is also used for predicting lexical complexity (Paetzold and Specia, 2016; Yimam et al., 2018; Desai et al., 2021; Shardlow et al., 2021a,b). This is since words with a high number of syllables can be hard to pronounce for some individuals (Mukherjee et al., 2016). L2 English speakers who are not yet familiar with the phonology of the target language, may subsequently find such words to be difficult to read and articulate (Mukherjee et al., 2016; Desai et al., 2021). Learners of English may perceive these words to be more complex than words with less syllables in comparison to L1 English speakers. Syllable count is normally obtained by counting the number of vowels within a target word (Desai et al., 2021).

#### 2.3.3 Character *N*-grams

Many languages do not share the same writing system or the same alphabet. This may lead to some L2 English speakers being unfamiliar with certain character combinations found in English. Thus, words made up of these unfamiliar character combinations are also likely to be considered more complex for a L2 English speaker than those words which have a similar appearance to words within their L1, i.e., cognate words. Certain character combinations may subsequently impact reading and understanding either as a consequence of being part of an acquired alphabet or simply being unfamiliar to the reader.

Character N-grams are often used to recognize those character combinations which may pose difficulty to a given reader (Desai et al., 2021; Shardlow et al., 2021b). We suspect that these differing character combinations may be identifiable when analyzing the bigrams and trigrams of the complex words annotated by L1 and L2 English speakers.

#### 2.3.4 Psycholinguistic features

Familiarity is among the most popular psycholinguistic feature for LCP (Paetzold and Specia, 2016; Yimam et al., 2018; Desai et al., 2021; Shardlow et al., 2021b). Obtained from the MRC Psycholinguistic Database (Wilson, 1988), familiarity is a measure of how well-known a target word is to an individual and was obtained through self-report from a group of 36 L1 English speaking university students (Gilhooly and Logie, 1980; Desai et al., 2021). Familiarity is related to another feature referred to as prevalence.

Prevalence is the percentage of annotators who know a target word (Brysbaert et al., 2019). It is produced by the following equation:


(3) $$\begin{array}{l} Prev\left(word\right)=\frac{Annotators\left(word\right)}{N} \end{array}$$

with the numerator being the number of annotators familiar with the word, and *N* being the total number of annotators. Brysbaert et al. (2019) has provided a dataset containing 62,000 English words and their respective prevalence ratings annotated by 221,268 L1 English speakers from the USA and UK.

Concreteness is another popular feature for LCP (Paetzold and Specia, 2016; Yimam et al., 2018; Desai et al., 2021; Shardlow et al., 2021b). It is defined as “the degree to which the concept denoted by a target word refers to a perceptible entity” (Brysbaert et al., 2013). Concreteness is also normally obtained through self-report. Brysbaert et al. (2013) have provided a dataset containing the concreteness ratings of 40,000 English words provided by 4,000 L1 English speakers located in the USA.

#### 2.3.5 Summary and hypotheses

In the preceding sections, we compare the above features' correlations with lexical complexity across multiple datasets created by differing sets of annotators: L1 and L2 English speakers. Zipfian, True, and document frequency, word length, syllable count, and character n-grams were computed manually, whereas familiarity, prevalence, and concreteness scores were extracted from the MRC Psycholinguistic Database (Wilson, 1988; Brysbaert et al., 2019, 2013), respectively, and then applied to each of the three datasets. We put forward several hypotheses.

**Hypothesis 1:** We suspect that strong correlations will exist between lexical complexity and word frequency, word length, syllable count, familiarity, prevalence, and concreteness, regardless of the type of annotator.
**Hypothesis 2:** We do, however, hypothesize that the strength of these strong correlations shall vary between datasets and their respective annotators.
**Hypothesis 3:** We predict that there shall be differences between the most complex bigrams and trigrams belonging to either set of annotators.

## 3 Data extraction and normalization

This study has extracted the English tokens without context and their corresponding continuous complexity values provided by the L1 English speaking annotators of the CompLex dataset (Shardlow et al., 2020), and the L2 English speaking annotators of the WCL dataset (Maddela and Xu, 2018), and the CERF-J wordlist (Tono, 2017). In total, 940 tokens were found to be shared among these datasets. However, 1 and 18 tokens were not matched with either prevalence or concreteness scores, respectively. These tokens were not considered within our final analysis of prevalence or concreteness and lexical complexity.

To compare L1 and L2 English speakers' complexity assignments, each dataset complexity values were normalized to a range between 0 and 1. Normalization was achieved through the following equation:


(4) $$\begin{array}{l} z_{i}=\frac{x_{i}-min\left(x\right)}{max\left(x\right)-min\left(x\right)} \end{array}$$

where *x*_*i*_ is the current complexity value, and min(x) and max(x) are the respective minimum and maximum values of the given likert scale range. Table 2 provides a snapshot of the 940 shared tokens along with their normalized complexity values.

**Table 2:** Example of 10 target words shared between the three datasets: CompLex, WCL, and CERF-J.

**Target word**	**CompLex**	**WCL**	**CERF-J**
Flour	0.17	0.17	0.20
Bulb	0.18	0.20	0.20
Kindness	0.19	0.26	0.40
Curse	0.19	0.20	0.20
Biology	0.30	0.37	0.40
Elite	0.34	0.31	0.60
Modification	0.31	0.54	0.60
Ideology	0.35	0.63	0.60
Equity	0.36	0.63	0.60
Infringement	0.40	0.70	0.60

Ranked from least to most complex per the L1 English speakers of the CompLex dataset.

## 4 Results

**Figure 1:**
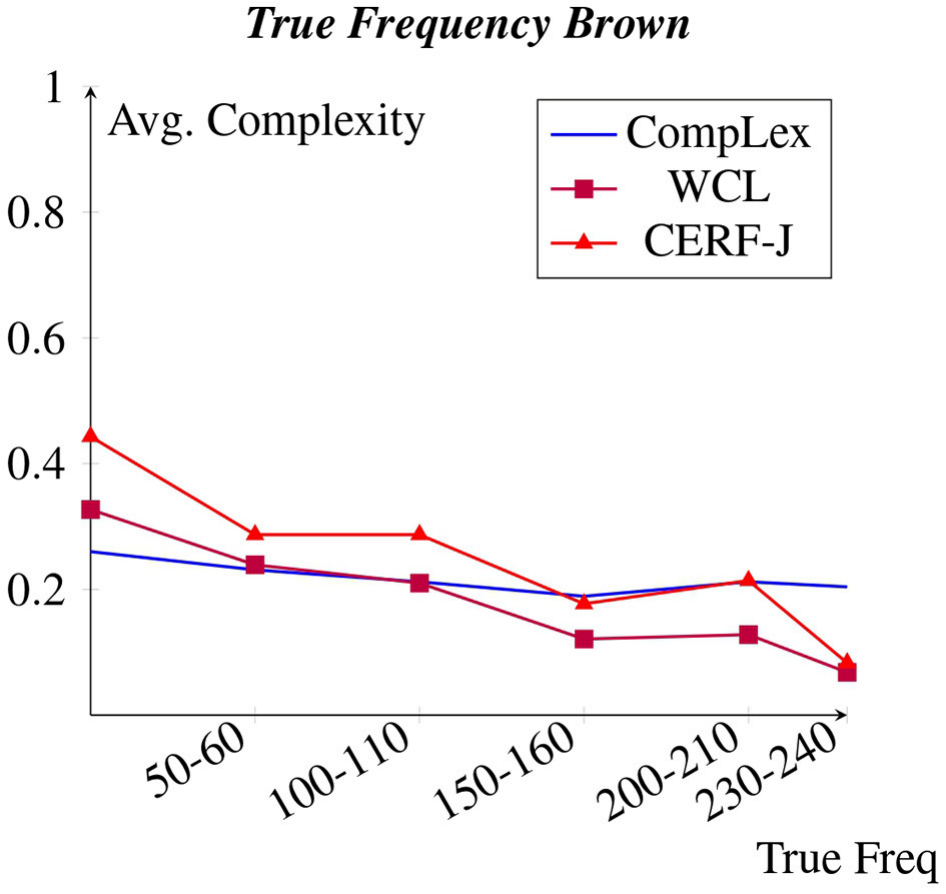
Avg. complexity per brown true freq.

**Figure 2:**
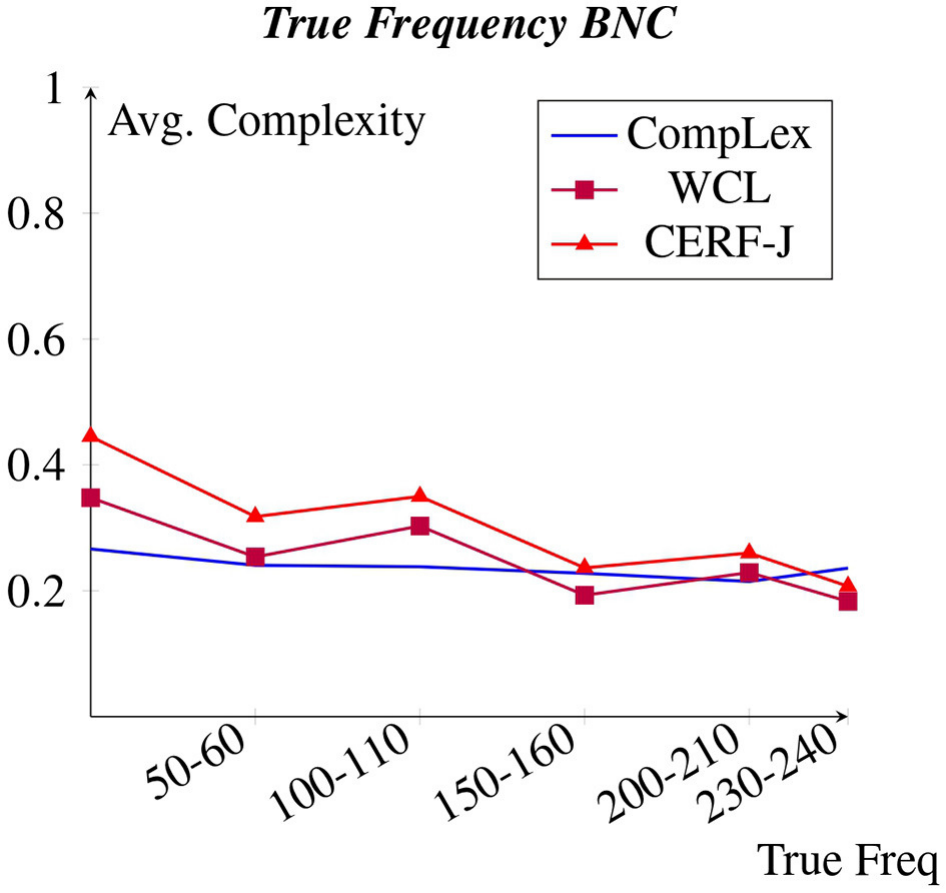
Avg. complexity per BNC true freq.

**Figure 3:**
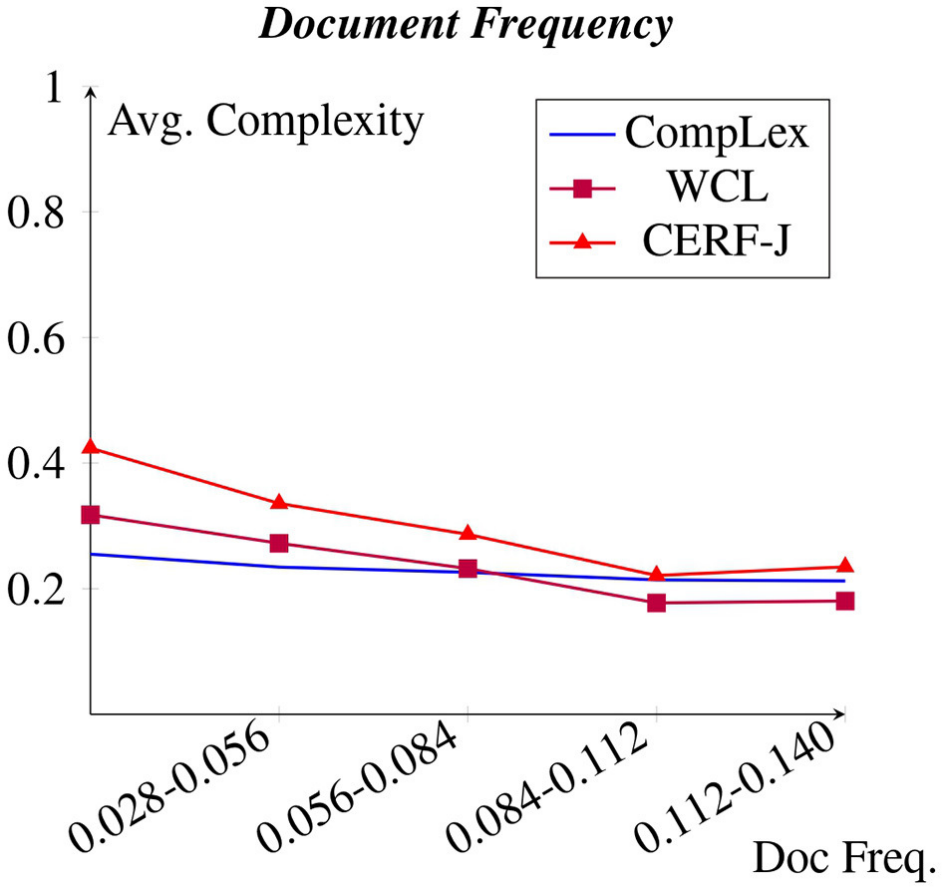
Avg. complexity per relative document frequency within the BNC.

**Figure 4:**
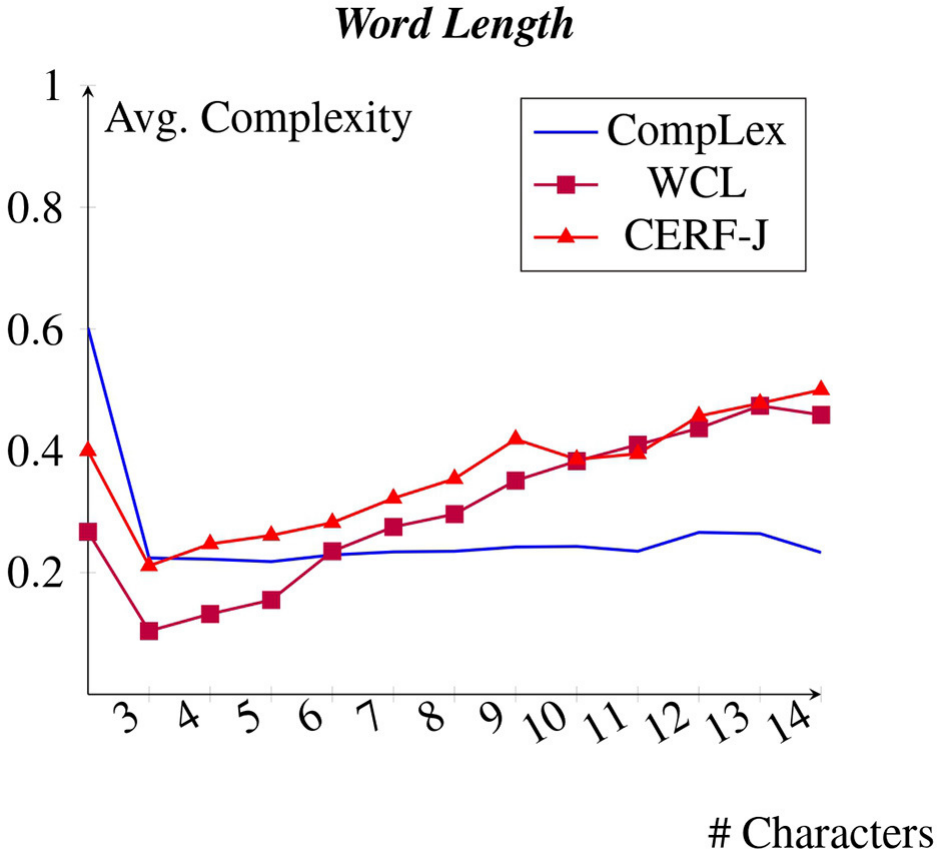
Avg. complexity per word length. within the BNC.

**Figure 5:**
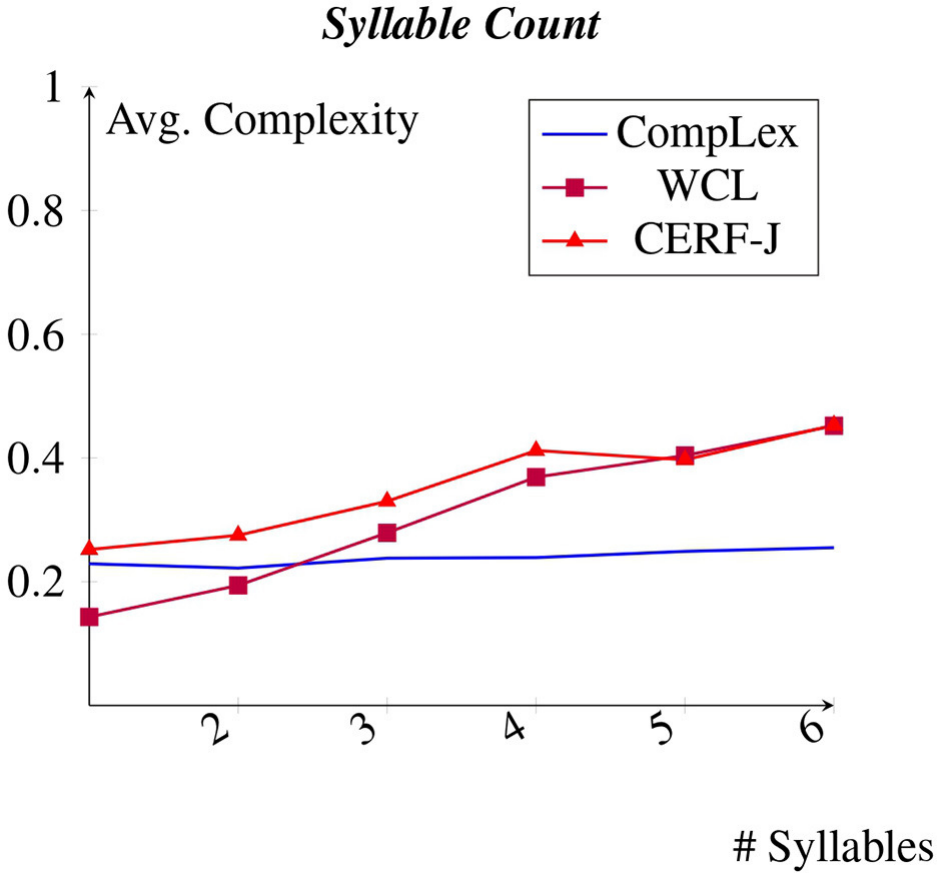
Average complexity per syllable.

**Figure 6:**
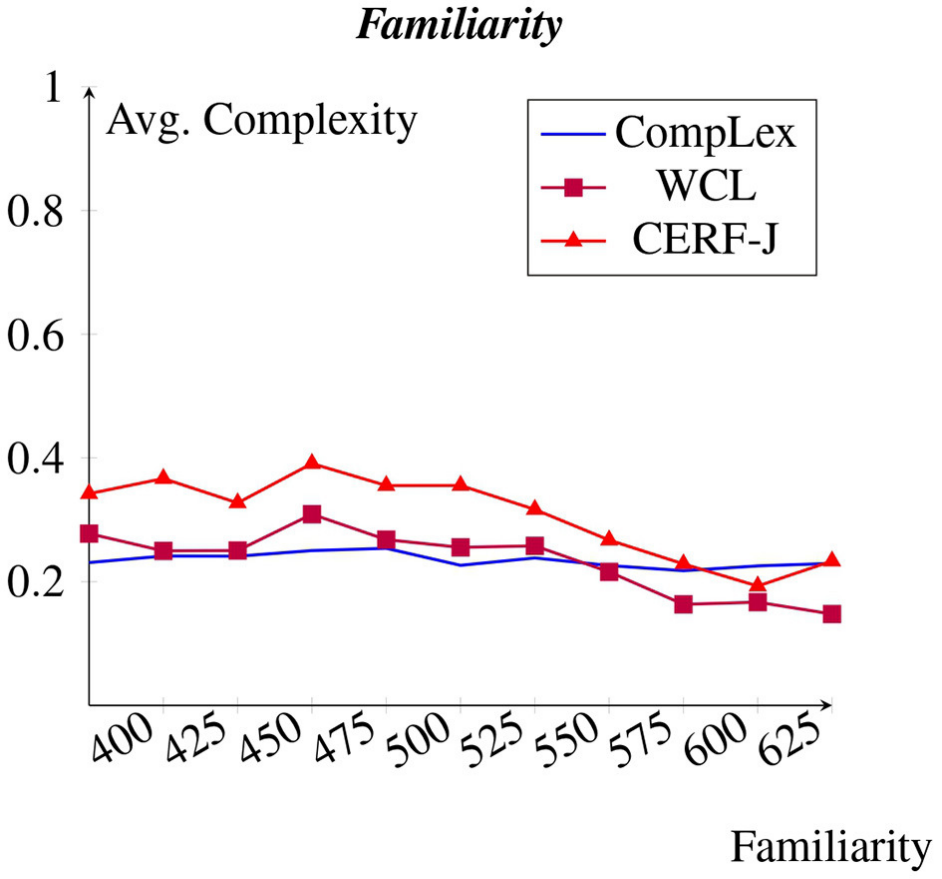
Avg. complexity per familiarity from the psycholinguistic datasbase (Wilson, 1988).

**Figure 7:**
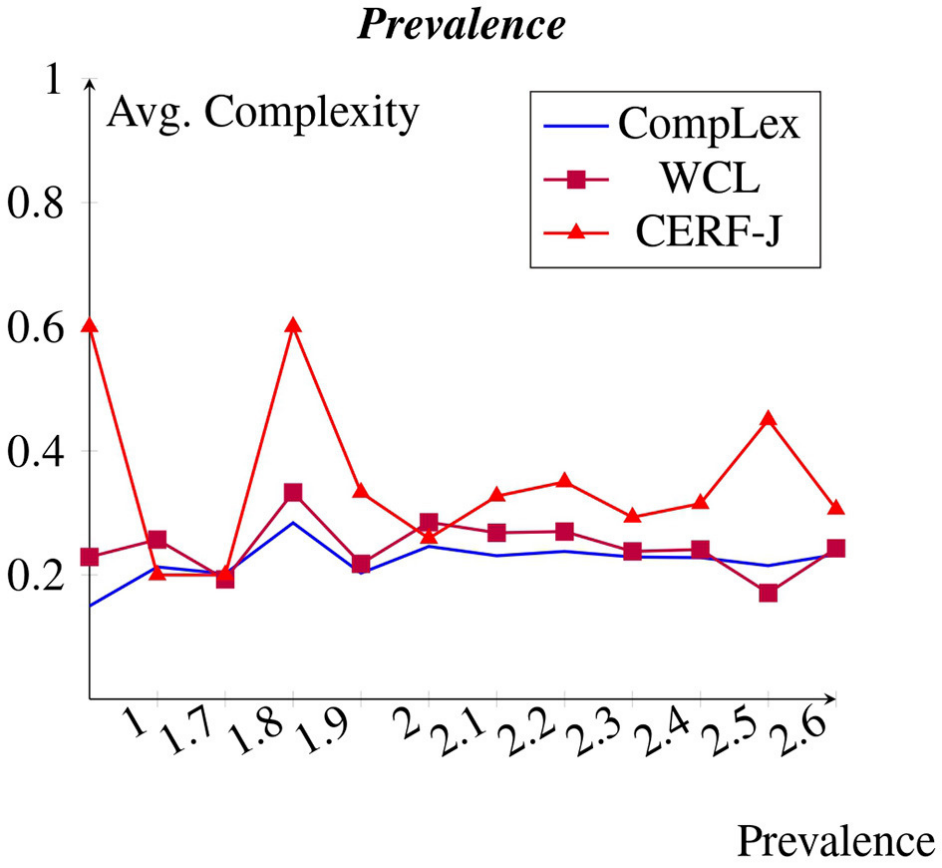
Avg. complexity per prevalence from Brysbaert et al. (2019).

**Figure 8:**
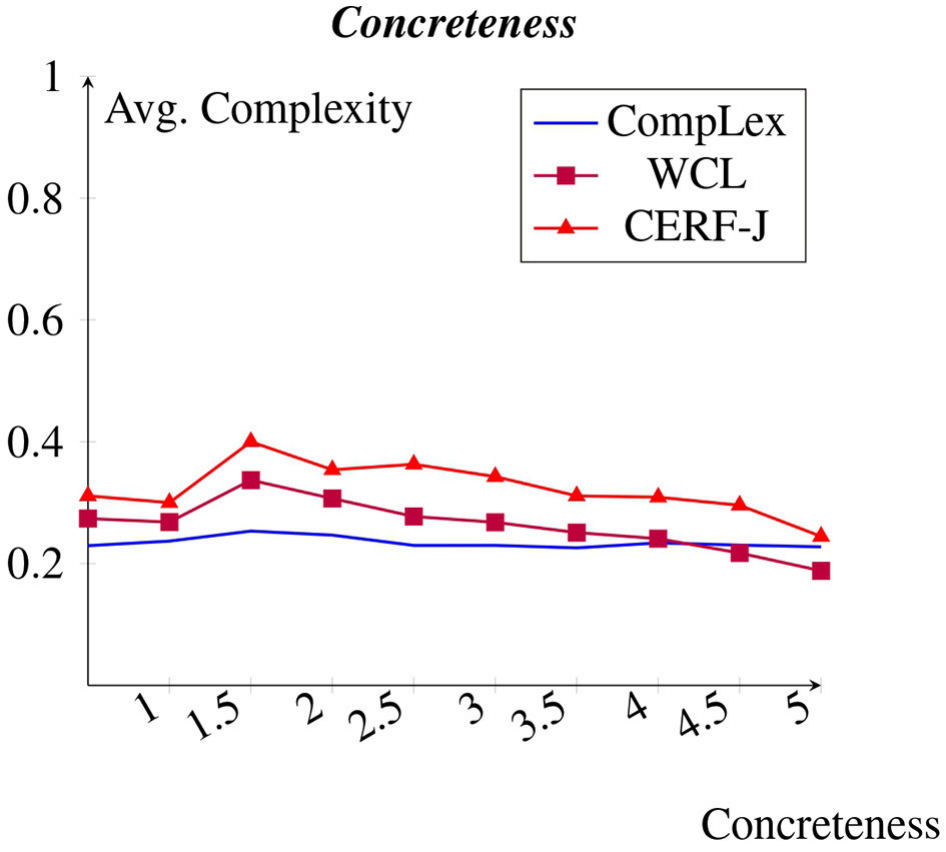
Average complexity per concreteness rating taken from Brysbaert et al. (2013).

The following sections compare the relationships between the chosen features and the normalized continuous complexity values made by either set of annotators. Figures 1–8 depict each features correlation to lexical complexity per dataset. The average lexical complexity values of L1 English speakers are shown in blue and have no symbol (CompLex), whereas those provided by L2 English speakers are represented by a purple square (WCL) and a red triangle (CERF-J).

### 4.1 Zipfian and true frequency

Figures 1, 2 display each datasets' average complexity values per token frequency within the Brown Corpus and BNC, respectively (Francis and Kucera, 1979). We predicted that in accordance to Zipf's Law, a negative correlation would exist between word frequency and complexity, with words of a higher frequency having been assigned lower complexity values. This would appear to be true for all datasets, particularly for the WCL dataset and the CERF-J wordlist. The 100 tokens with the lowest Zipfian frequencies, including such words as “*valentine*”, “*genetics*”, and “*functionality*”, were on average +0.20 and +0.27 more complex than the 100 tokens with the highest Zipfian frequencies, including such words as “*may*”, “*first*”, and “*new*”, within the WCL dataset and the CERF-J wordlist, respectively. This negative correlation is also supported by examining these 100 tokens' True frequencies both in regards to the Brown and BNC datasets. The 100 tokens with the lowest True frequencies per the Brown corpus were on average +0.13 and +0.19 more complex than those 100 tokens with the highest True frequencies within the WCL dataset and the CERF-J wordlist, respectively. Furthermore, an average decrease of −0.0012 per +10.00 increase in True Frequency was observed for those complexity assignments belonging to the WCL dataset and −0.0019 for those belonging to the CERF-J wordlist. An average decrease of −0.0065 and −0.0099 per +10.00 increase in True Frequency was likewise observed per the BNC for the WCL dataset and the CERF-J wordlist, respectively.

The CompLex dataset alternatively depicted a less strong negative correlation between word frequency and complexity. For most instances, the frequency of a given token did not appear to have a great influence on its assigned complexity. The 100 least frequent tokens were found to be on average only +0.06 more complex in regards to their Zipfian frequencies and on average only +0.04 more complex per the Brown corpus and +0.05 more complex per the BNC in regards to their True frequencies. The 100 most frequent tokens were on average rated to be −0.15 less complex by the CompLex dataset's L1 annotators than they were by the WCL dataset's L2 annotators and the CERF-J wordlist in regards to their True frequency. *P*-values of 0.0004 and 0.0035 suggest that there is a significant difference between the complexities of the 100 most and least frequent tokens of either set of annotators, respectively. In addition, a less impressive average decrease of −0.00008 in complexity using the Brown corpus and a decrease of −0.0013 using the BNC per +10.00 increase in True Frequency was also observed for the CompLex dataset.

### 4.2 Document frequency

Figure 3 shows a snapshot of the relative document frequency of target words found within the BNC. It was discovered that those target words with a relative document frequency >0.140 exhibited a similar complexity. However, a decrease in assigned complexity can be seen between relative document frequencies of 0 to 0.140. This is in parallel with the negative correlation shown between assigned complexity and word frequency for CompLex, WCL, and the CERF-J wordlist within Section 4.1. An average decrease of −0.0017 for CompLex, −0.0054 for WCL, and −0.0075 for the CERF-J wordlist was seen per +0.006 increase in relative document frequency. As such, the L2 annotators of the WCL and CERF-J wordlist seem to be more affected by the frequency of a word compared to the L1 annotators of the CompLex dataset.

### 4.3 Word length

Figure 4 depicts each dataset average complexity values per word length. We predicted that longer words would be less familiar and more difficult to learn for set of annotators and consequently would be rated with higher complexity values. This was seen to be true across all datasets. Each dataset demonstrated a positive correlation between word length and complexity. Tokens with 3–7 characters, including such words as “*day*”, “*men*”, and “*may*”, were rated to be on average −0.15 less complex than tokens with 10–14 characters, examples being “*management*”, “*international*”, and “*relationship*”. On average, a +0.03 increase in complexity was observed per every additional character across all datasets.

The CompLex dataset appears to show a less strong positive correlation between word length and complexity compared with the WCL dataset and the CERF-J wordlist. Words with 4–7 characters were assigned with an average complexity of 0.22. Words with 10–14 characters were rated as having an average complexity of 0.25. Therefore, on average, words with 10–14 characters were perceived to be +0.03 more complex than those with 4–7 characters for the CompLex dataset's L1 English speaking annotators. In comparison, the L2 English speaking annotators of the WCL dataset and the CERF-J wordlist, respectively, assigned words with 10–14 characters with complexity values that were on average +0.25 and +0.18 greater than those with 4–7 characters. The L1 English speaking annotators of the CompLex dataset have subsequently interpreted long words of 10–14 characters to be on average −0.19 less complex than compared with the L2 speaking annotators of the WCL dataset and the CERF-J wordlist. A *p*-value of 0.0002 between the complexities assignments of 10–14 character words belonging to either set of annotators, confirms that this difference is significant.

### 4.4 Syllable count

Figure 5 displays each datasets average complexity values per number of syllables within a given token. The WCL dataset and the CERF-J wordlist showed a positive correlation between assigned complexity and a target word's number of syllables. The CompLex dataset, however, demonstrated no such positive correlation. Instead, the CompLex dataset showed little to no fluctuation in complexity between 1 and 5 syllable words. For every additional syllable in this range, the CompLex dataset shows an extremely small increase in complexity of +0.004. Thus, no real change in complexity was observed. The WCL dataset and the CERF-J wordlist, on the other hand, showed incremental increases in complexity between 1 and 5 syllables by +0.06 and +0.04, respectively. This further proves that the number of syllables contained within a target word are less important for L1 English speakers when it comes to rating that word's complexity, whereas for L2 English speakers, an increased number of syllables may result in greater word difficulty. This is especially true if that word contains 5 or more syllables. However, a *p*-value of 0.077 indicates that the complexity assignments given to 1 to 5 syllable words are not significantly different between the two sets of annotators. As such, these observations should/must be verified on a larger sample of L1 and L2 English speakers.

### 4.5 Character *N*-grams

The 10 most complex bigrams and trigrams with a frequency greater than 10 found among the 940 shared tokens are presented within Table 3. Several observations suggest that certain derivations, character combinations, or morphemes, increase the perceived complexity of target words for L2 English speakers, yet have no affect on complexity assignment for L1 English speakers.

**Table 3:** Average complexities and frequency of the top 10 most complex bigrams and trigrams across the three datasets.

**Bigram**	**CompLex**	**CWI**	**CERF-J**	**Frequency**	**Trigram**	**CompLex**	**CWI**	**CERF-J**	**Frequency**
***io***	**0.25**	**0.37**	**0.40**	**118**	***ati***	**0.25**	**0.40**	**0.42**	**54**
***ti***	**0.24**	**0.37**	**0.40**	**111**	*nce*	0.25	0.39	0.41	31
*co*	0.25	0.35	0.40	60	*men*	0.24	0.48	0.42	29
*ic*	0.25	0.35	0.38	37	*con*	0.25	0.36	0.41	24
*ns*	0.25	0.37	0.45	24	*ran*	0.26	0.39	0.43	13
*rs*	0.22	0.3	0.40	15	*tra*	0.26	0.37	0.39	13
*va*	0.27	0.37	0.47	15	*ica*	0.27	0.44	0.5	11
*ue*	0.25	0.33	0.40	14	*fic*	0.27	0.42	0.44	11
*mm*	0.25	0.41	0.36	10	*cat*	0.25	0.38	0.47	11
*ef*	0.28	0.32	0.48	10	***nes***	**0.22**	**0.34**	**0.45**	**10**

Bigrams and trigrams of interest are in bold. Ordered most frequent to least.

The trigram “*nes*” within the WCL dataset and the CERF-J wordlist was found to be in more complex words than it was within the CompLex dataset. This trigram was found among 10 of the 940 shared words, and was part of such words as “*awareness*”, “*thickness*”, “*kindness*”, “*weakness*”, and “*righteousness*”. Its associated words were on average assigned a complexity value (a difficulty rating) of 0.34 for the WCL dataset and a complexity value of 0.45 for the CERF-J wordlist by the original annotators of the datasets. Within the CompLex dataset, however, these words were rated with an average complexity value of 0.22 and thus were on average rated as being −0.18 less complex. This may indicate that for L2 English speakers, target words with the derivational suffix “*-ness*”, are considered to be more complex than they are for L1 English speakers.On the other hand, the trigram “*nes*” was also found in words, such as “*Chinese*” or “*honest*”, and given the small sample size, further investigation was needed to verify this finding. As such, we calculated the average complexity assignment for all of the words with the derivation “*-ness*” found within each dataset, including those words which were not shared. In total, the CompLex dataset was found to contain 82 words with the “*-ness*” derivation with an average complexity of 0.26, whereas the WCL dataset and the CERF-J wordlist contained 39 and 52 words with the “*-ness*” derivation with average complexity ratings of 0.34 and 0.48, respectively.

The bigrams “*io*” and “*ti*”, and the trigram “*ati*” belonged to words with noticeably higher complexity values within the WCL dataset and the CERF-J wordlist than they did within the CompLex dataset. The bigrams “*io*” and “*ti*”, were part of 118 and 111 words, respectively, and the trigram “*ati*” was part of 54 words of the shared 940 words. Many of these words had all three n-grams as they contained the suffix “*-tion*”, for example: “*isolation*”, “*separation*”, “*discrimination*”, “*classification*”, and “*communication*”. Those words that contained the bigrams “*io*” and “*ti*” had an average complexity value of 0.37 for the WCL dataset and an average complexity value of 0.4 for the CERF-J wordlist. The trigram “*ati*” was part of words with an average complexity of 0.4 for the WCL dataset and an average complexity of 0.41 for the CERF-J wordlist. In comparison, the bigrams “*io*” and “*ti*”, and the trigram “*ati*”, were found to have been associated with average complexity values of 0.25, 0.24, and 0.25 for the Complex dataset, respectively. The suffix “*-tion*” would, therefore, appear to be present within words that are on average +0.15 more complex for L2 English speakers than they are for L1 English speakers.

Words without derivation, hence root words (lemmas), appeared to be less complex than those words with an derivational prefix or suffix, regardless of the dataset or annotator. 741 of the 940 shared words were root words. On average, root words were found to have complexity values of 0.23, 0.22, and 0.28 for the CompLex dataset, the WCL dataset, and the CERF-J wordlist, respectively. The 199 remaining derivational words, had average complexity values of 0.24, 0.36, and 0.42 for the CompLex dataset, the WCL dataset, and the CERF-J wordlist, respectively. As such, the 741 root words appeared to be on average -0.01, -0.1, and -0.13 less complex across the three datasets when compared to those words with derivation. Derivation therefore would appear to universally increase the complexity of a target word.

L2 English speakers have also appeared to have found derivational word forms more troublesome than L1 English speakers. This is since L2 English speakers have assigned words with the derivations: “*-ness*” or “*-tion*” with greater complexity values than compared to the L1 English speakers of the CompLex dataset, as detailed above. However, there were only a few instances were both root and derivational word forms were shared across the three datasets, such as “*complex*” and “*complexity*”, “*effect*” and “*effectiveness*”, “*portion*” and “*proportion*”, “*relation*” and “*relationship*”, “*action*” and “*interaction*”, and so forth. The average differences between these root words and their derivational forms were complexities values of +0.01, −0.17, and −0.16 for the CompLex dataset, the WCL dataset, and the CERF-J wordlist, respectively. This suggests that the prior assumption is correct. For example, for the L2 English speaking annotators of the WCL dataset and the CERF-J wordlist, these root words were on average −0.16 to −0.17 less complex than compared to the L1 English speaking annotators of the CompLex dataset. Nevertheless, without further root and derivational word pairs, this finding is inconclusive.

### 4.6 Familiarity and prevalence

As expected, a negative correlation was observed between familiarity and complexity (see Figure 6). However, this was only seen within the WCL dataset and the CERF-J wordlist. For these datasets, an average increase in perceived complexity was found of +0.002 per every −10 decrease in familiarity. The complexity assignments of the CompLex dataset did not demonstrate this trend. Instead, familiarity appeared to have had no affect on complexity assignment, with complex and non-complex words depicting similar or varying degrees of familiarity. For example, the 100 most familiar words found within the WCL dataset and the CERF-J wordlist had an average complexity of 0.16 and 0.22, respectively, whereas their 100 least familiar words had an average complexity of 0.27 and 0.31, respectively. This resulted in a difference of +0.11 for the WCL dataset and a difference of +0.09 for the CERF-J wordlist. In contrast, the 100 most and least familiar words found within the CompLex dataset depicted average complexity values of 0.22 and 0.22, respectively amounting to a range of −0.003. The difference between the 100 least familiar words' complexity assignments between the two sets of annotators was also found to be significant with a *p*-value less than 0.001. In turn, familiarity has little to no impact on complexity within the CompLex dataset than in comparison to the WCL dataset and the CERF-J wordlist.

A less strong negative correlation was observed between prevalence and complexity across all of the datasets than expected (see Figure 7). However, for the CompLex dataset this correlation was even less emphatic. Words with little to no prevalence appeared to have been assigned with similar complexity values to those words which were rated as being highly prevalent. The 100 most prevalent tokens, including such words as “*party*”, “*building*”, and “*morning*”, were assigned an average complexity of 0.22 and 0.27, whereas the 100 least prevalent tokens, made up of such words as “*honor*”, “*economy*”, and “*market*”, were rated an average complexity of 0.27 and 0.36 for the WCL dataset and the CERF-J wordlist, respectively. The 100 most prevalent tokens were therefore −0.05 and −0.09 less complex in comparison to the 100 least prevalent tokens for these datasets. For the CompLex dataset, however, the 100 most and least prevalent tokens were assigned respective average complexities of 0.229 and 0.233. Therefore, this marks a less impressive decrease in complexity by −0.004 for the 100 most prevalent tokens. A *p*-value of 0.048 marks a slight significant difference between the assigned average complexities of the 100 least prevalent tokens between both sets of annotators. It would, therefore, appear that both familiarity and prevalence are good indicators of complexity, but only for complexity assignments made by L2 English speakers. This is since L2 English speakers demonstrated a far stronger positive correlation between these two features and complexity, than in comparison to L1 English speakers.

### 4.7 Concreteness

A negative correlation was observed between concreteness and complexity (see Figure 8). This negative correlation is again more prominent within the WCL dataset and the CERF-J wordlist. Those tokens which were assigned concreteness values of 5, marking them as highly concrete, for example “*tree*”, “*sand*”, “*house*”, “*chair*”, and “*water*”, were on average assigned complexity values of 0.19, 0.14, and 0.22 in the CompLex dataset, the WCL dataset, and the CERF-J wordlist, respectively. Those tokens which were given concreteness values of 0, identifying them as highly abstract, for instance “*attitude*”, “*online*”, “*complex*”, “*righteousness*”, and “*impact*”, were on average assigned complexity values of 0.23, 0.27, and 0.31 across the three datasets, respectively. As such, for every −1.00 decrease in concreteness, the WCL dataset and the CERF-J wordlist depicted an average increase in complexity by +0.02. The CompLex dataset, on the other hand, showed a less impressive increase in complexity by +0.006 per −1.00 decrease in concreteness. Furthermore, a *p*-value of 0.03 marks that this difference is significant. As such, concreteness appears to have more of an effect on perceived lexical complexity for L2 English speakers, than it does for L1 English speakers.

## 5 Discussion

### 5.1 Statistical features and complexity

A negative correlation was found between word and document frequency and complexity for the CompLex dataset, the WCL dataset, as well as the CERF-J wordlist. This was unsurprising given that previous studies (Paetzold and Specia, 2016; Yimam et al., 2018; Desai et al., 2021; Shardlow et al., 2021b) have demonstrated that word frequency is a good baseline indicator of lexical complexity. However, the strength of this negative correlation varied between L1 and L2 English speakers. The WCL dataset and the CERF-J wordlist, being annotated by L2 English speakers, depicted a significantly stronger negative correlation than in comparison to the CompLex dataset that was annotated by L1 English speakers.

The same finding was also observed in regards to word length. The WCL dataset and the CERF-J wordlist both showed a strong positive correlation between the number of characters in a word and that word's assigned complexity, with the CompLex dataset again showing a significantly less strong correlation between the two. Word length has also been proven to be a good baseline indicator of lexical complexity (Paetzold and Specia, 2016; Yimam et al., 2018; Desai et al., 2021; Shardlow et al., 2021b). Nevertheless, since that both word frequency and word length vary in their correlations with lexical complexity between L1 and L2 English speakers, it can be assumed that uncommon words of a greater length are far more complex for L2 English speakers than they are for L1 English speakers.

A possible explanation is that L2 English speakers are far less likely to be exposed to and thus be familiar with uncommon and long English words and would subsequently rate these words as being more complex than in comparison with L1 English speakers. Furthermore, English words that are over 6 characters long, are likely to contain a high number of syllables and would generally be hard to pronounce and learn given their length. This would likely increase the perceived complexity of such words, especially for someone who is unacquainted with English vocabulary or phonology. For instance, words such as “*righteousness*”, “*vanity*”, “*conscience*”, “*nucleus*”, and “*genetics*” received greater complexity values by L2 English speakers than compared to L1 English speakers. These tokens are all jargon that is specific to the religious or academic genre per the CompLex dataset (Shardlow et al., 2020). They were also of considerable length compared with other less complex tokens.

### 5.2 A phonological feature and complexity

A positive correlation was observed between syllable count and complexity for both the WCL dataset as well as the CERF-J wordlist. However, this was not the case for the CompLex dataset. This may support the assumption that words with a high number of syllables are especially hard for L2 English speakers. However, this cannot be certain, since a *p* > 0.05 indicates no significant difference between the complexity assignments of 1–5 syllable words belonging to either set of annotators. In spite of this, if L2 English speakers were to find an increase number of syllables difficult to articulate and/or process, then this may again be due to a possible unfamiliarity with English phonology, or, a more likely explanation is a phenomenon known as cross-linguistic influence.

Cross-linguistic influence is where an individual's L1 has an active effect on their L2 production. This phenomenon results in a variety of production errors. For instance, a L1 Chinese speaker whom is unfamiliar with English pluralization may incorrectly use the singular form of an English target word in a plural setting. This is because Chinese does not use inflection to dictate pluralization (Yang et al., 2017). Cross-linguistic influence can subsequently be linked to differing perceptions of lexical complexity, with some demographics finding specific English words, word forms, or pronunciations, to be more or less complex than others, depending on their L1.

Certain vowels, diphthongs, or phonological patterns are specific to certain languages and are not present in English, or are unique to English yet are not found in other languages. As a consequence, those words with a high number of syllables are more likely to contain troublesome phonemes which may be prone to cross-linguistic influence. An example of this, is the English /*i*ə/ diphthong, as found in *b*eer, *p*ier, and *w*ea*ry* (Enli, 2014). For Chinese L2 English speakers, this particular diphthong is hard to pronounce, since there is no equivalent sound in Chinese Mandarin. Therefore, the lack of a similar sounding Chinese phoneme causes those English words that contain the /*i*ə/ diphthong to be hard for Chinese L2 English speakers to articulate and potentially learn (Enli, 2014). As a result, Chinese annotators may rate such words as being more complex than compared with those English words that they find easier to articulate and that have a less number of syllables. Thus, a greater number of syllables increases the likelihood of troublesome phonemes prone to cross-linguistic influence. This may explain the observed positive correlation between number of syllables and L2 English speaker's perceived lexical complexity depicted in Figure 3.

### 5.3 Morphological features and complexity

#### 5.3.1 “*-ness*” suffix

The “*-ness*” suffix is used to transform a noun, or a root word, to a countable noun, such as changing “*thick*” to “*thickness*”, or “*kind*” to “*kindness*”. It is also derivational in that it can be used to change the meaning of a word to denote a related but separate concept, such as “*weak*” to “*weakness*” as in “*he was weak*” to “*his weakness is*”. Interestingly, words that contained the “*-ness*” suffix were rated as being significantly more complex by the L2 English speakers of the WCL dataset and the CERF-J wordlist, than in comparison to the L1 English speakers of the CompLex dataset.

#### 5.3.2 “*-tion*” suffix

The “*-tion*” suffix is used to transform verbs to abstract nouns, such as transforming “*isolate*” to “*isolation*”, or “*discriminate*” to “*discrimination*”. Words that contained the “*-tion*” suffix were also found to be more complex for L2 English speakers than L1 English speaker in the respective datasets.

#### 5.3.3 Root words

Unlike words with the above derivations, those words without derivation appeared to be universally less complex than in comparison to those with derivation. This being across all annotators and datasets.

Prior research has attempted to explain the neural processing of L1 derivations (Kimppa et al., 2019). However, less research has been conducted on how speakers process derivations within their L2 (Kimppa et al., 2019). Several hypotheses have been put forward that attempt to explain why production errors are caused in connection to L2 derivation and in turn, why L2 English speakers may perceive such words, such as those words with the suffix “*-ness*” or “*-tion*”, to be more complex than root words without derivation (Gor, 2010).

L2 processing is believed to be more cognitively demanding. It is theorized to require more cognitive resources than L1 processing. As result, it can lead to delayed response time and production errors even among highly proficient L2 speakers (McDonald, 2006; Clahsen and Felser, 2018). McDonald (2006), conducted several experiments and found that L1 English speakers produced the same grammatical errors as L2 English speakers when in a stressful and high processing environment, such as dealing with noisy data or given a short time to respond. McDonald (2006) concluded that L2 speakers must therefore experience high cognitive demand whenever they process their L2. Hopp (2014) goes on to explain this further. He demonstrated that increased cognitive demand during L2 processing results in there being insufficient resources for syntactic processing. This may subsequently explain why such forms as “*-ness*” and “*-tion*” as well as other derivations, are perceived to be more complex for L2 than compared with L1 English speakers. Inadequate working memory may cause L2 English speakers to have difficulty with decoding these derivations, whereas L1 English speakers having less cognitive load, can do so with ease.

The shallow-structure hypothesis (Clahsen and Felser, 2006a,b, 2018), is unlike the above explanations. Alternatively, this hypothesis infers that a difference in L2 processing is responsible for differences in complexity assignment between L1 and L2 English speakers, rather than an increased cognitive load and inadequate cognitive resources. It suggests that L2 learners rely more on lexical and semantic information than syntactic cues when attempting to derive the meaning of a given sentence. In other words, the shallow-structure hypothesis puts forward that an L2 learner's syntactic representations are often “shallower and less detailed” in their L2. It hypothesizes that this is a result of direct form-function mapping, memorizing a particular form of a given L2 word, rather than ascertaining that form from learned L2 syntactic rules (Dowens and Carreiras, 2006). As such, the shallow-structure hypothesis is also different from cross-linguistic influence, since the former is concerned with differences in L1 and L2 processing, whereas the latter is entirely a consequence of L1 influence on L2; however, some cross-over between the two does occur (Clahsen and Felser, 2018). If the shallow-structure hypothesis were to be true, then this would explain why such “*-ness*” and “*-tion*” words were perceived to be more complex for L2 in comparison to L1 English speakers. For instance, L2 English speakers may only be able to recall those word forms which they are familiar with, having memorized the word: “*awareness*”, rather than learning the uses of the derivation “*-ness*”. L1 English speakers, on the other hand, may be better equipt to infer the meaning of an unseen word form, based on their prior syntactic knowledge of that derivation: “*-ness*”. In turn, such words would appear less complex for L1 than L2 English speakers.

### 5.4 Psycholinguistic features and complexity

#### 5.4.1 Familiarity and prevalence

It was expected that both familiarity and prevalence would demonstrate a negative correlation with complexity, with higher familiarity and prevalence ratings resulting in reduced perceived lexical complexity. Results showed this to be true, but only for the WCL dataset and the CERF-J wordlist. The CompLex dataset showed no such trend between familiarity and complexity, with only a small negative correlation between prevalence and complexity being present.

A possible explanation may be found in the annotation process of the provided familiarity and prevalence ratings taken from the MRC psycholinguistic database (Wilson, 1988) and Brysbaert et al. (2019)'s dataset, respectively. Both of these datasets acquired their familiarity and prevalence ratings from a set of L2 English speaking annotators of mixed proficiency. It can, therefore, be expected that a stronger correlation would exist between other L2 English speakers' complexity ratings and the familiarity and prevalence ratings provided by these datasets than in comparison to those complexity ratings provided by L1 English speakers. Nevertheless, this does not diminish the importance of familiarity and prevalence in regards to lexical complexity. Another potential explanation is that L1 and L2 English speakers are more or less familiar with differing words as previously mentioned in Section 5.1. L1 English speakers may be aware of regional varieties, dialects, or vernacular. For instance, “*wild*” could be considered to have vernacular connotations in British and American English. This may explain why similar vernacular words were rated as having high familiarity and low complexity by L1 English speakers, yet low familiarity and high complexity by L2 English speakers. Various studies (Desai et al., 2021; Shardlow et al., 2021b) have also proven that there is correlation between familiarity, prevalence and lexical complexity. As such, our finding that the CompLex dataset did not reflect a negative correlation between these features and lexical complexity, may be a direct result of the poor generalizability of the MRC psycholinguistic database (Wilson, 1988) and Brysbaert et al. (2019)'s dataset toward L1 English speakers, rather than there being no such correlation.

#### 5.4.2 Concreteness

Concreteness was found to negatively correlate greater with the complexity assignments of L2 English speakers than compared with those belonging to L1 English speakers. It is well-documented that concrete nouns are learned before, processed faster, and recalled more easily than abstract nouns (Altarriba and Basnight-Brown, 2011; Vigliocco et al., 2018). The same can be said for L2 English learners. Altarriba and Basnight-Brown (2011), conducted a Stroop color-word test. They measured their L2 English speaking participants reaction time to various concrete and abstract nouns. They discovered that concrete nouns were responded to significantly faster than abstract nouns. Martin and Tokowicz (2020) discovered a similar finding. They tested L1 English speakers ability at learning L2 concrete and abstract nouns. It was recorded that concrete nouns “were responded to more accurately than abstract nouns” (Martin and Tokowicz, 2020). Mayer et al. (2017) conducted a vocabulary translation task during fMRI scans on L2 English learners. They found that L2 concrete and abstract nouns elicited the same responses as L1 concrete and abstracts nouns and subsequently concluded that L2 nouns are likely to be prone to the same concreteness effects as L1 nouns.

The above studies exemplify a phenomenon known as the concreteness effect. This phenomenon refers to the negative correlation between a noun's level of concreteness and its overall acquisition difficulty and processing time. It can subsequently be used to describe the negative correlation found within this study between concreteness and perceived lexical complexity. This is since those words which are learned later and take longer to process would likely be considered more complex. There are several possible explanations for this phenomenon.

The context availability hypothesis (Martin and Tokowicz, 2020), states that differences in concrete and abstract noun complexity is due to the differing contexts in which these words are found. For instance, a concrete noun, such as “*chair*” is likely to be more common and appear in more contexts than the abstract noun “*communism*”. The dual-coding theory (Paivio, 2006) as well as the different organizational frameworks theory (Crutch et al., 2009), suggest that the human mind represents concrete and abstract words differently. Concrete nouns, for instance “*chair*”, are believed to be encoded with visual cues in accordance to their real world manifestation, such as “*cushion*”, “*chair leg*”, or “*arm rest*”. Abstract nouns, however, for example “*communism*”, are represented as concepts, having less emphatic visual identifiers with more symbolic associations, “*red*”, or “*hammer and sickle*”.

## 6 Spelling error classification

### 6.1 Use case

Spelling errors are symptomatic of lexical complexity. Complex words are more likely to be misspelt than non-complex words. An individual that is familiar with a word is more likely to know that word's orthography than in comparison to an unfamiliar and unknown word (Paola et al., 2014). With this in mind, we hypothesized that the above differences in perceived lexical complexity between L1 and L2 English speakers could be used to differentiate between spelling errors made by these two target populations.

It is well-documented that L2 English speakers make different types of spelling errors compared to L1 English speakers (Napoles et al., 2019). However, the connection between spelling error and lexical complexity as defined within the field of natural language processing has been left fairly unexplored (North et al., 2022). A doctoral thesis by Wu (2013) looked into the relationship between self-reported word frequency, familiarity, and morphological complexity with spelling error. A sample of 220 5th to 7th grade L1 English speakers were taken from American schools. Results indicated a strong negative correlation between word frequency and spelling error for 7th grade students, a slight negative correlation between familiarity and spelling error for all students, and a positive correlation between morphological complexity and spelling error that decreased with age.

It is plausible that words that are frequently misspelt may exhibit the same features that mark words as being complex. The previous analysis in Section 5 indicates that features such as word frequency, length, syllable count, familiarity and prevalence, and concreteness are more greatly correlated with L2 than L1 English speakers' perception of lexical complexity. We, therefore, used these features to train several binary machine learning (ML) classifiers to distinguish between spelling errors made by L1 and L2 English speakers. This was done to test the validity of our previous observations and to provide a use case for our findings within TS and CALL technologies.

### 6.2 Dataset and models

Our original analysis was conducted on a sample of 940 shared-tokens. To obtain the best possible performance, we included all instances from the CompLex dataset (3,144) and a equal number of instances from the WCL and CERF-J wordlist (3,144) for our train set.

The test set was obtained from the dataset introduced by Napoles et al. (2019), being a different dataset from those used within the above analysis. This dataset was created for grammatical error correction and provides spelling mistakes made by L1 and L2 English speakers. It houses 1,984 and 1,936 sentences extracted from formal Wikipedia articles written by L1 English speakers and a collection of student essays written by L2 English speakers, respectively. Napoles et al. (2019) asked a set of 4 trained annotators to examine each sentence and identify grammatical and spelling errors. We selected 396 of the spelling errors made by L1 and 287 of the spelling errors made by L2 English speakers. We used these spelling errors as our test set having labeled each instance with a corresponding L1 or L2 spelling error label (shortened to L1 or L2).

We trained a total of five binary ML classifiers. These included a Random Forest (RF), Support Vector Classifier (SVC) and a Naive Bayes (NB) model, as well as two baseline models in the form of a majority classifier (MC), and a random classifier (RC). These models were trained on the aforementioned features described in Section 2.3 with the exception of character n-grams. Given the size of our test set, models were unable to draw meaningful correlations between character n-grams and spelling error. Average age-of-acquisition (AoA) was also included as an additional feature. AoA is defined as the age at which a word's meaning is first learned (Desai et al., 2021). It was calculated by averaging the AoAs provided by Brysbaert and Biemiller (2017). Each model was also trained on four feature sets, as explained below. These feature sets contained a combination of different features based on their individual performances:

1. **A**. Contained several of what are considered the baseline statistical features of LCP (Shardlow et al., 2020), being word length, syllable count, and frequency.
2. **B**. Consisted of the best individually performing features of frequency, prevalence and concreteness.
3. **C**. Expanded feature set B to also include AoA.
4. **D**. Contained all features.

### 6.3 Performance

Models were assessed on their macro f1-scores since there was an equal distribution of class labels within the train and test sets. Marco f1-score being the average f1-score achieved per-class label. Despite the RF, SVC, and NB models having achieved similar performance to the RC and MC baseline models, increases in performance were observed when certain features were taken into consideration. Table 4 lists all model performances for each feature and feature set.

**Table 4:** Macro f1-scores produced by each feature and feature set ordered from highest to lowest score on feature set C, being the best performing feature set.

	**Features**						**Feature sets**			
**Model**	**Frequency**	**Word length**	**Syllable count**	**Prevalence**	**Concreteness**	**AoA**	**A**	**B**	**C**	**D**
RF	**0.577**	0.489	0.422	**0.552**	**0.572**	**0.570**	0.509	0.562	**0.611**	0.559
SVC	0.430	0.513	0.498	0.503	0.478	0.453	0.510	0.506	0.530	0.531
NB	0.506	0.513	0.498	0.506	0.499	0.453	0.501	0.506	0.523	0.526
RC	0.521	0.487	0.499	0.496	0.483	0.529	0.488	0.510	0.495	0.514
MC	0.367	0.367	0.367	0.367	0.367	0.367	0.367	0.367	0.367	0.367

Best performances are in bold. Dash lines separate baseline models: RC and MC, and feature set A, from non-baseline experiments.

**Figure 9:**
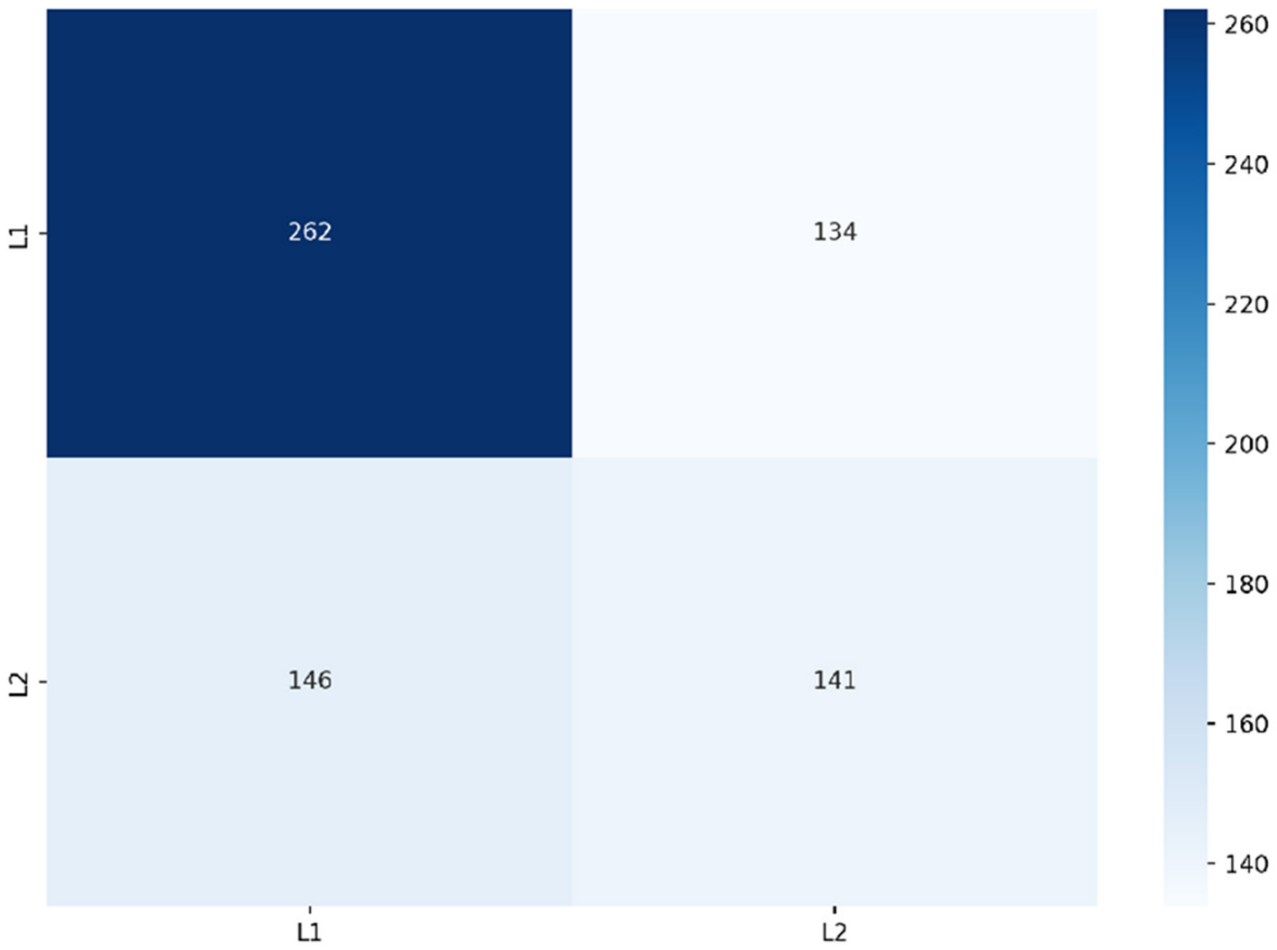
Predictions of RF trained on frequency.

Frequency was found to improve our RF model's performance to a macro f1-score of 0.577 surpassing the highest performance achieved by our baseline models. Figure 9 depicts the class predictions of our RF when trained on frequency. Our RF model was able to use frequency to correctly predict a large number of the L1 English speakers' spelling errors, whilst simultaneously being less successful with predicting spelling errors made by L2 English speakers.

**Figure 10:**
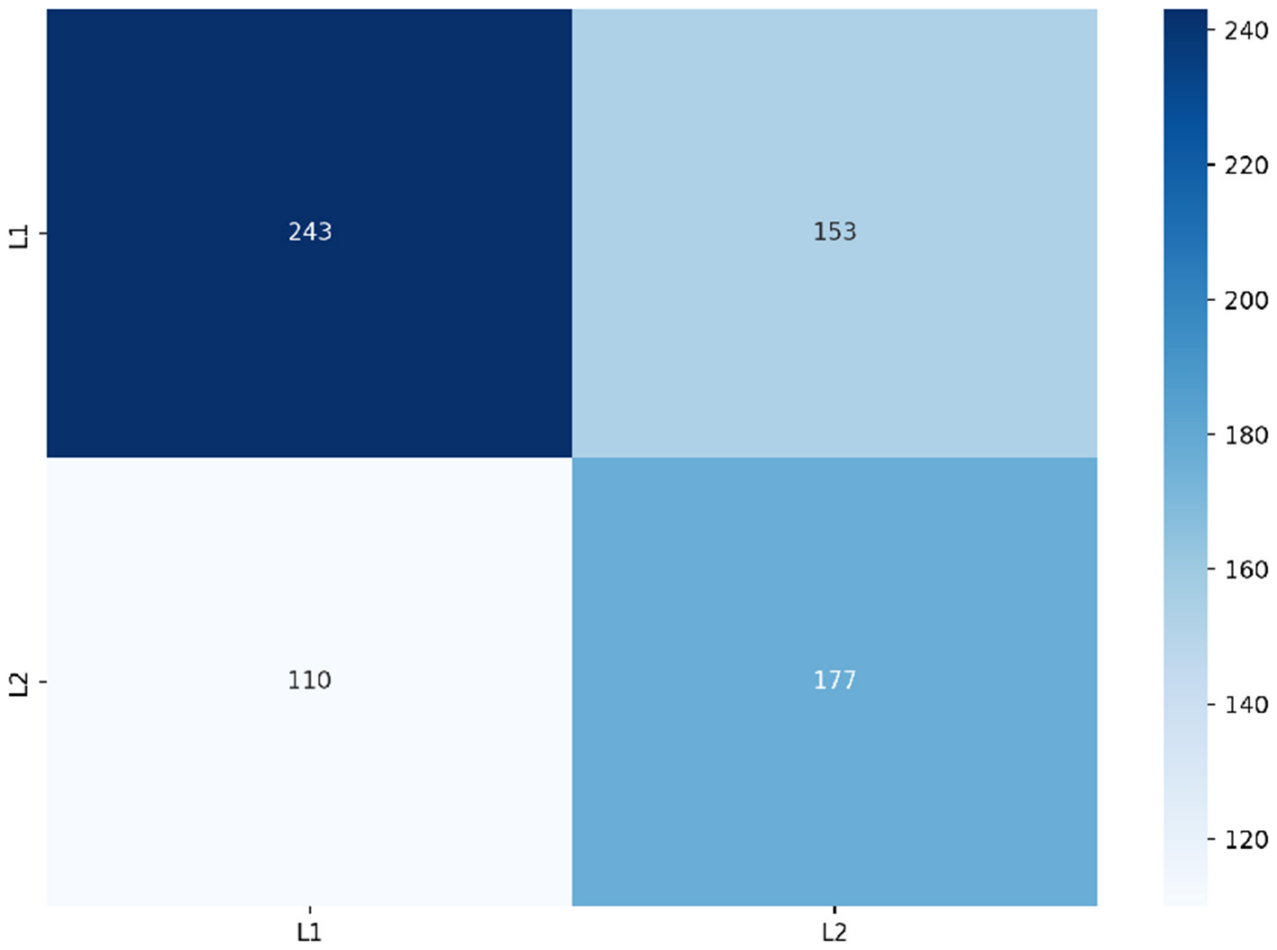
Predictions of RF trained on set C.

The psycholinguistic features of prevalence, concreteness and AoA likewise improved our RF's performance achieving macro f1-scores of 0.552, 0.572, and 0.570, respectively. However, in contrast to frequency, these features improved our RF's ability to predict L2 English speakers' spelling errors. Figure 10 shows the class prediction of our RF when trained on feature set C comprising these psycholinguistic features plus frequency. After having been trained on this feature set, our RF was able to correctly predict a larger number of spelling errors made by L2 English speakers alongside those instances already correctly predicted as belonging to L1 English speakers by our previous frequency-based RF. Feature set C resulted in our RF achieving it's highest performance with a macro f1-score of 0.611.

To determine whether our RF's performance using feature set C was statistically significant to that achieved by our RC baseline, predictions using feature set C were generated ten times. A *t*-test was then applied to the f1-scores achieved by our RF and RC models. The *p*-value obtained is lower than 0.05 indicating that the performance of our RF trained on feature set C is statistically significant compared to our RC baseline.

Feature sets B and D surprisingly performed less well than feature set C having produced slightly worse macro f1-scores of 0.562 and 0.559, respectively. We contribute this to feature set C's inclusion of AoA. Feature set B did not include this feature, whereas feature set D included all features which likely convoluted class boundaries.

### 6.4 Transferable features

Frequency, prevalence, concreteness, and AoA are features that can be gained from LCP datasets and then used to train a binary classifier for predicting spelling errors made by L1 or L2 English speakers. Contrary to our previous observation in Section 5, frequency was found to be indicative of L1 English speakers' spelling errors more so than spelling errors made by L2 English speakers. This is likely a result of the strong correlation observed between frequency and lexical complexity, regardless of the target population (Wu, 2013). Prevalence, concreteness, and AoA, on the other hand, were able to predict more spelling errors made by L2 English speakers. This supports our previous finding. It suggests that words that are less prevalent, less concrete, and are learned at a later age are prone to being misspelt by L2 English speaker's, hence are likely to be considered more complex by this demographic.

## 7 Conclusion and outlook

This study aimed to discover whether sizable differences exist between the lexical complexity assignments of L1 and L2 English speakers. The complexity assignments of 940 shared tokens were extracted and compared from three LCP datasets: the CompLex dataset (Shardlow et al., 2020), the WCL dataset (Maddela and Xu, 2018), and the CERF-J wordlist (Tono, 2017). It was found that word frequency, length, and syllable count had a greater effect on perceived lexical complexity for L2 English speakers than they did for L1 English speakers. Various derivations: “*-ness*” and “*-tion*” increased lexical complexity for L2 English speakers more so than L1 English speakers. Root words were seen to be universally less complex by comparison with their derivational forms. Familiarity and prevalence influenced lexical complexity for L2 English speakers, yet for L1 English speakers only prevalence had an effect. Concreteness was found to predict lexical complexity mainly for L2 English speakers. However, a less emphatic correlation was also observed for L1 English speakers.

In an attempt to explain these findings, we have briefly introduced the reader to a number of potential explanations taken from applied linguistics. These range from familiarity (Desai et al., 2021; Shardlow et al., 2021b), cross-linguistic influence (Lee and Yeung, 2018b; Maddela and Xu, 2018), greater cognitive load in L2 processing (McDonald, 2006; Hopp, 2014), the shallow-structure hypothesis (Clahsen and Felser, 2006b, 2018), the context availability hypothesis (Martin and Tokowicz, 2020), the dual-coding theory (Paivio, 2006), and the different organizational frameworks theory (Crutch et al., 2009).

Our findings were lastly applied to the task of automatically classifying spelling errors made by L1 and L2 English speakers. It was found that frequency obtained from the CompLex dataset was able to predict spelling errors made by L1 English speakers. Familiarity, prevalence, and concreteness, on the other hand, obtained from the WCL dataset and the CERF-J wordlist, were able to predict more spelling errors made by L2 English speakers. As such, we demonstrate that several features ascertained from LCP datasets are transferable and that several of our findings are generalizable. We aim to use the differences between L1 and L2 English speakers perception of lexical complexity to better develop personalized LCP systems, in turn, aiding future TS and CALL applications.

## Data availability statement

Publicly available datasets were analyzed in this study. This data can be found at: https://github.com/MMU-TDMLab/CompLex.

## Author contributions

KN: writing, data analysis, experimentation, and theory. MZ: writing, theory, proof reading, and supervision.
